# Low Rate of Detection of Mucosal High-Risk-Type Human Papillomavirus in Korean Patients with Extragenital Bowen's Disease and Squamous Cell Carcinoma, Especially in Digital Cases

**DOI:** 10.1155/2013/421205

**Published:** 2013-08-19

**Authors:** Hye-Rim Park, Kwang Ho Kim, Soo Kee Min, Jinwon Seo, Dong Hoon Kim, Mi Jung Kwon

**Affiliations:** ^1^Department of Pathology, College of Medicine, Hallym University Sacred Heart Hospital, 896 Pyungchon-Dong, Dongan-Ku, Anyang 431-070, Republic of Korea; ^2^Department of Dermatology, College of Medicine, Hallym University Sacred Heart Hospital, 896 Pyungchon-Dong, Dongan-Ku, Anyang 431-070, Republic of Korea

## Abstract

Human papillomavirus (HPV) infection has been demonstrated in some of the nonmelanoma skin cancers as well as in precancerous lesions. Multiple infections of mucosal high-risk HPV may contribute to the onset of digital Bowen's disease through, if any, digital-genital transmission. We screened for the presence of the mucosal HPV DNA in patients with extragenital Bowen's disease (*n* = 30), squamous cell carcinoma (*n* = 11), bowenoid papulosis (*n* = 9), verrucous carcinoma (*n* = 1), actinic keratosis (*n* = 5), and basal cell carcinoma (*n* = 5). We used a PANArray HPV Genotyping Chip for high-risk and low-risk mucosal types. Genotyping data was confirmed using a conventional direct DNA sequencing method. Two cases of extragenital Bowen's disease were positive for types 16 and 33 of mucosal HPV, respectively. None of the squamous cell carcinoma cases were positive. Neither patients with digital Bowen's disease (*n* = 5) nor those with squamous cell carcinoma (*n* = 3) showed any mucosal high-risk HPV. Mucosal high-risk HPV DNA was confirmed in 5 (55.6%) of the 9 patients with bowenoid papulosis. HPV 16 was most prevalent (*n* = 3), while the DNA of HPVs 35 and 67 was detected in one sample for each of the two types. Our study demonstrated that two (6.7%) of the patients with 30 extragenital Bowen's disease were positive for types 16 and 33 of mucosal HPV, respectively. HPVs belonging to the mucosal high-risk group may participate in the development of extragenital Bowen's disease. However, we could not find any relationship between the mucosal high-risk HPV and Bowen's disease or squamous cell carcinoma in the fingers.

## 1. Introduction

Human papillomavirus (HPV) is increasingly recognized as an important human carcinogen. HPVs can be divided into cutaneous and mucosal groups according to their target sites. HPV infection was demonstrated in some of the nonmelanoma skin cancers including squamous cell carcinoma, verrucous carcinoma, and precancerous lesions including epidermodysplasia verruciformis (EV), Bowen's disease, and bowenoid papulosis [1–3]. While more than 20 types of EV HPVs are known, cutaneous cancers are predominantly associated with HPV types 5 and 8 and much less frequently with HPV types 14, 17, 20, and 47 [4]. The pathogenic role of beta HPVs in nonmelanoma skin cancer has not yet been completely understood and the literature indicates that they might at least be cofactors in the development of certain cutaneous squamous cell carcinomas. However, the role of HPVs in the pathogenesis of basal cell carcinoma in immunocompetent individuals is unclear [5].

Among mucosal high-risk HPVs, HPVs 16 and 18 are known as major causal factors for cervical cancer. Bowen's disease and squamous cell carcinoma in the genital area are generally thought to be associated with mucosal high-risk HPVs, as in cervical cancer [6]. In a prevalence study, the odds ratio for nonmelanoma skin cancer in patients who were DNA-positive for the high-risk mucosal HPV types 16, 31, 35, and 51 was 59, with normal skin as a control [7]. These findings suggest that persistent infections of the skin with high-risk genital HPV types identified as significant risk factors for cervical cancer may also represent a risk factor for nonmelanoma skin cancer in a nonimmunosuppressed population.

Recent studies have shown that mucosal high-risk HPVs are related to the development of extragenital Bowen's disease [1, 3, 7–10]. However, the detection rate and spectrum of HPVs in extragenital Bowen's disease are not yet agreed upon, and it is not clear to what extent HPV is involved in its pathogenesis [1, 3, 7–10]. Interestingly, multiple infections of mucosal high-risk HPV may contribute to the onset of digital Bowen's disease through, if any, finger-genital transmission [1].

In the present study, we screened for the presence of the DNA of mucosal HPVs in patients with extragenital Bowen's disease, squamous cell carcinoma, or bowenoid papulosis. For comparison, cases of verrucous carcinoma, actinic keratosis, and basal cell carcinoma were included. In particular, we focused on any possible detection of mucosal high-risk HPVs in digital cases of Bowen's disease and squamous cell carcinoma.

## 2. Materials and Methods

*Cases. *We examined a total of 61 formalin-fixed and paraffin-embedded resection and biopsy specimens from patients with extragenital Bowen's disease (*n* = 30), squamous cell carcinoma (*n* = 11), bowenoid papulosis (*n* = 9), verrucous carcinoma (*n* = 1), actinic keratosis (*n* = 5), and basal cell carcinoma (*n* = 5). We also included five cases of normal skin as a control. All patients were immunocompetent. As shown in Table 1, the sites of Bowen's disease were the finger (*n* = 5), hand and wrist (*n* = 7), buttock (*n* = 3), abdomen (*n* = 3), back (*n* = 5), chest (*n* = 1), thigh (*n* = 3), ankle (*n* = 1), arm (*n* = 1), and scalp (*n* = 1). The sites of squamous cell carcinoma were the finger (*n* = 3), thigh (*n* = 2), ankle (*n* = 1), face (*n* = 4), and scalp (*n* = 1). The study was approved by the institutional review board.

### 2.1. Preparation of DNA Samples

DNA extraction was performed as described previously [11]. Genomic DNA was extracted from the formalin-fixed, paraffin-embedded tissue sections of each sample using a Magna Pure LC instrument. Briefly, 10 *μ*m paraffin sections were gently mixed with 800 *μ*L of xylol and 400 *μ*L of ethyl alcohol absolute by inverting the tube several times. The supernatant was discarded after brief centrifugation and the pellet was washed with 1 mL of ethyl alcohol absolute. The pellet was dried for 10 min at 55°C after the removal of the supernatant. The tissue pellet was vortexed with 80 *μ*L of a tissue lysis buffer (Roche diagnostics GmbH, Mannheim, Germany) and 20 *μ*L of proteinase K, followed by overnight incubation at 55°C. The digested samples were loaded into the Magna Pure LC instrument (Roche diagnostics GmbH). All DNA samples were stored at −70°C before use.

### 2.2. HPV Genotype Assay Using the PANArray HPV Kit

The PANArray HPV Genotyping Chip (Panagene, Daejeon, South Korea) detects 19 high-risk and 13 low-risk mucosal HPV types. HPVs 16, 18, 26, 31, 33, 35, 39, 45, 51, 52, 53, 56, 58, 59, 66, 68, 69, 70, and 73 are high-risk HPV types, and HPVs 6, 11, 32, 34, 40, 42, 43, 44, 54, 55, 62, 81, and 83 are low-risk types. Analyses were performed according to the manufacturer's instructions (PCR amplification, hybridization, washing, and scanning) [12].

*Sequencing. *Genotyping data obtained from HPV chips were confirmed using a conventional direct DNA sequencing method, as described in a previous report [13]. Type-specific PCR was performed using primers appropriate for each genotype. The primers were designed with reference to the polymorphic regions of the HPV L1 sequences. Two microliter aliquots of PCR products were amplified using type-specific primers.

## 3. Results

Two (6.7%) of the 30 extragenital Bowen's disease specimens were positive for types 16 and 33 of mucosal high-risk HPV, respectively. The locations of these positive cases were wrist and buttock, respectively. There were no clinically significant findings in mucosal high-risk HPV-positive cases compared to negative ones. The two positive patients were immunocompetent. None of the 11 squamous cell carcinoma samples were positive. Patients with digital Bowen's disease (*n* = 5) or squamous cell carcinoma (*n* = 3) did not show any mucosal high-risk HPV. Meanwhile, mucosal high-risk HPV DNA was confirmed in 5 (55.6%) of the 9 patients with bowenoid papulosis. HPV 16 was most prevalent (*n* = 3), while HPVs 35 and 67 were detected in one sample each. The locations were the pubis in 3 cases and the penis in 2 cases. Cases of verrucous carcinoma, actinic keratosis, and basal cell carcinoma were all negative. Cases of normal control skin were also all negative. No positive results were found in mucosal low-risk HPVs.

## 4. Discussion

The present study demonstrated that the frequency of mucosal high-risk HPV in patients with extragenital Bowen's disease was 6.7%, which was similar to the frequency seen in the report by Zheng et al. (7%) [3]. In contrast, Nakajima et al. [1] reported the frequency to be 14%, which was twice as high as our result. HPVs 16 and 33 were detected in patients with Bowen's disease in our study, which is also similar to the report by Zheng et al. [3]. They also confirmed the distribution of HPV DNA among most nuclei of the tumor cells but in none of the cells of the adjacent normal skin by *in situ* hybridization [3]. In a study by Nakajima et al., HPV 16 is more predominant than HPVs 58 and 33 in Bowen's disease cases [1]. On the other hand, HPV 33 was the most prevalent mucosal high-risk HPVs type detected in nonmelanoma skin cancers in the United States and Germany [7]. This strongly suggests that mucosal high-risk HPV might be involved in the development of some small proportions of extragenital Bowen's disease lesions.

We determined whether there might be characteristic clinical or histological findings among the two Bowen's disease patients with mucosal high-risk HPV. Macroscopically and histologically, however, there were no significant differences between high-risk HPV-positive and negative patients. They had no other histories of systemic disease or malignancy. Notably, the locations of the lesions were the buttock and wrist. The buttock is near the anogenital area that is the main target site of mucosal HPV. Although the detection rates of HPV in patients with Bowen's disease in the hands and feet, especially in the fingers and toes, were reported to be high [1, 10, 14–16], our cases (5 cases in the fingers and 5 cases in the hand) were all negative. Most of the reported patients with digital Bowen's disease who harbored mucosal high-risk HPVs had lesions in the proximal or lateral sides of the fingers [1]. In contrast, Bowen's disease of the trunk and limbs showed a low frequency of mucosal high-risk HPV. Notably, 3 cases of Bowen's disease in the fingers were double-positive for mucosal and cutaneous high-risk HPVs [1]. Multiple infections of mucosal and cutaneous high-risk HPV may contribute to the onset of digital Bowen's disease through, if any, finger-genital transmission.

Digital squamous cell carcinoma presents a diagnostic challenge because of its relatively rare occurrence and mimicry of benign conditions. Although low-risk HPV subtypes are commonly associated with benign digital verrucae, digital squamous cell carcinoma can be associated with high-risk, oncogenic HPV subtypes. In this study, patients with digital squamous cell carcinoma (*n* = 3) did not show any mucosal high-risk HPV, and all patients were immunocompetent. These results are in contrast with those of a previous report which indicated relatively high prevalences of mucosal high-risk HPVs in digital squamous cell carcinoma [17, 18]. This discrepancy may be due to differences in patient immunocompetence or sexual behavior, including possible finger-genital transmission. The DNA detection method or small sample size of digital cases should also be considered as other possibilities of discrepancy. Some limitations to the laboratory methods were reported in the use of type-specific PCR primers, *in situ* hybridization, or Southern blot analysis and resulted in the biased detectability of individual types [7]. 

Kreuter et al. [19] demonstrated that all the cases that were positive for mucosal high-risk HPVs were periungual squamous cell carcinoma rather than the proximal or lateral parts of the fingers. Gormley et al. [20] reported 7 patients, including 4 HIV-positive patients, who presented with 10 lesions of digital squamous cell carcinoma *in situ*. Multiple high-risk oncogenic subtypes were found, including HPVs 16, 33, 51, and 73. Their case series highlights the diversity of oncogenic HPV types that may be associated with digital squamous cell carcinomas although the majority of reports linking HPV and digital squamous cell carcinomas have implicated the HPV 16 subtype. Because the high rate of recurrence of digital squamous cell carcinomas may be a result of the persistence of oncogenic HPV at the margin of resection, aggressive treatment of individual lesions and of genital reservoirs for HPV in patients and their sexual partners is warranted [20]. 

In our study, cases of verrucous carcinoma, actinic keratosis, and basal cell carcinoma were all negative for mucosal high-risk HPV. In basal cell carcinoma, 16 different types of beta HPV were found and the most common types were HPVs 107, 100, and 15, all belonging to the beta HPV species 2 [5]. However, according to a prospective case-control study of basal cell carcinoma, HPV does not seem to play a fundamental role in the pathogenesis of either nodular or superficial basal cell carcinoma [21]. The presence of HPV appears to be more related to actinic damage and possibly to an alteration of the barrier function associated with aging [21]. Four beta HPV species 2 (HPVs 107, 110, 111, and FA75) were more common in patients with actinic keratosis than in healthy skin, but the prevalence and viral loads were low [22].

## 5. Conclusions

Our study demonstrated that two (6.7%) of the 30 patients with extragenital Bowen's disease were positive for types 16 and 33 of mucosal HPV, respectively. HPVs belonging to the mucosal high-risk group may participate in the development of some extragenital Bowen's diseases. However, we could not find any relationship between the mucosal high-risk HPV and Bowen's disease or squamous cell carcinoma in the fingers.

## Figures and Tables

**Table 1:** List of patients with Bowen's disease, bowenoid papulosis, and squamous cell carcinoma in our study.

Diagnosis	Patient	Age/sex	Location	HPV type detected
Bowen	1	47/M	Thumb, right	
Bowen	2	64/M	5th finger, left	
Bowen	3	52/M	4th finger, left	
Bowen	4	43/M	Thumb, right	
Bowen	5	50/M	4th finger, left	
Bowen	6	61/F	Hand	
Bowen	7	61/F	Hand	
Bowen	8	69/M	Hand	
Bowen	9	80/F	Hand	
Bowen	10	53/F	Hand	
Bowen	11	55/M	Wrist	HPV 33
Bowen	12	83/M	Wrist	
Bowen	13	70/F	Buttock	HPV 16
Bowen	14	48/M	Buttock	
Bowen	15	52/F	Buttock	
Bowen	16	56/M	Abdomen	
Bowen	17	57/F	Abdomen	
Bowen	18	61/M	Abdomen	
Bowen	19	45/F	Back	
Bowen	20	56/F	Back	
Bowen	21	81/F	Back	
Bowen	22	73/F	Back	
Bowen	23	49/F	Back	
Bowen	24	75/F	Chest	
Bowen	25	73/F	Thigh	
Bowen	26	69/F	Thigh	
Bowen	27	63/F	Thigh	
Bowen	28	56/F	Ankle	
Bowen	29	86/F	Arm	
Bowen	30	89/F	Scalp	

BP	31	27/M	Penis	HPV 16
BP	32	34/M	Pubis	HPV 35
BP	33	42/M	Penis	
BP	34	23/M	Penis	
BP	35	38/M	Penis	
BP	36	38/M	Penis	HPV 16
BP	37	47/M	Scrotum	
BP	38	37/F	Pubis	HPV 67
BP	39	60/F	Pubis	HPV 16

SCC	40	57/M	4th finger, right	
SCC	41	46/M	Thumb, left	
SCC	42	64/M	5th finger, left	
SCC	43	76/F	Thigh	
SCC	44	63/F	Thigh	
SCC	45	56/F	Ankle	
SCC	46	88/F	Temple	
SCC	47	92/F	Cheek	
SCC	48	91/F	Cheek	
SCC	49	76/M	Eyelid	
SCC	50	38/F	Scalp	

BP: bowenoid papulosis; SCC: squamous cell carcinoma.
